# Structural basis of peptide recognition by the angiotensin-1 converting enzyme homologue AnCE from *Drosophila melanogaster*

**DOI:** 10.1111/febs.12038

**Published:** 2012-11-22

**Authors:** Mohd Akif, Geoffrey Masuyer, Richard J Bingham, Edward D Sturrock, R Elwyn Isaac, K Ravi Acharya

**Affiliations:** 1 Department of Biology and Biochemistry, University of BathUK; 2 Faculty of Biological Sciences, School of Biology, University of LeedsUK; 3 Institute of Infectious Disease and Molecular Medicine, University of Cape TownSouth Africa

**Keywords:** angiotensin-1 converting enzyme, crystal structure, kinetics, natural peptides, substrate recognition

## Abstract

Human somatic angiotensin-1 converting enzyme (ACE) is a zinc-dependent exopeptidase, that catalyses the conversion of the decapeptide angiotensin I to the octapeptide angiotensin II, by removing a C-terminal dipeptide. It is the principal component of the renin-angiotensin–aldosterone system that regulates blood pressure. Hence it is an important therapeutic target for the treatment of hypertension and cardiovascular disorders. Here, we report the structures of an ACE homologue from *Drosophila melanogaster* (AnCE; a proven structural model for the more complex human ACE) co-crystallized with mammalian peptide substrates (bradykinin, Thr^6^–bradykinin, angiotensin I and a snake venom peptide inhibitor, bradykinin-potentiating peptide-b). The structures determined at 2-Å resolution illustrate that both angiotensin II (the cleaved product of angiotensin I by AnCE) and bradykinin-potentiating peptide-b bind in an analogous fashion at the active site of AnCE, but also exhibit significant differences. In addition, the binding of Arg–Pro–Pro, the cleavage product of bradykinin and Thr^6^– bradykinin, provides additional detail of the general peptide binding in AnCE. Thus the new structures of AnCE complexes presented here improves our understanding of the binding of peptides and the mechanism by which peptides inhibit this family of enzymes.

**Database:**

The atomic coordinates and structure factors for AnCE–Ang II (code 4AA1), AnCE–BPPb (code 4AA2), AnCE–BK (code 4ASQ) and AnCE–Thr6–BK (code 4ASR) complexes have been deposited in the Protein Data Bank, Research Collaboratory for Structural Bioinformatics, Rutgers University, New Brunswick, NJ (http://www.rcsb.org/)

**Structured digital abstract:**

- AnCE cleaves Ang I by enzymatic study (View interaction)
- Bradykinin and AnCE bind by x-ray crystallography (View interaction)
- BPP and AnCE bind by x-ray crystallography (View interaction)
- AnCE cleaves Bradykinin by enzymatic study (View interaction)
- Ang II and AnCE bind by x-ray crystallography (View interaction)

## Introduction

Peptidyl dipeptidase A is a highly glycosylated ectoenzyme that cleaves dipeptides from the C-terminus of a broad range of oligopeptides. The mammalian enzyme is also known as angiotensin-1 converting enzyme (ACE; a monomeric, membrane-bound, zinc- and chloride-dependent enzyme, EC 3.4.15.1) after the important role of endothelial ACE in the processing of angiotensin I (Ang I) to angiotensin II (Ang II), a powerful vasoconstrictor 1. The identification and biochemical characterization of the Ang I convertase led directly to the development of ACE inhibitors that are in widespread clinical use for the treatment of not only hypertension, but also heart failure and other cardiovascular and renal diseases 2–4. A characteristic that was not appreciated during the development of the first generation of ACE inhibitors was that endothelial (or somatic) ACE comprised two highly similar protein domains (N- and C-domains) linked by a short peptide with each domain possessing a catalytic centre with peptidyl dipeptidase activity. Although both active sites are promiscuous, they display certain substrate preferences 5,6. In mice, the C-domain is primarily responsible for the *in vivo* conversion of Ang I to Ang II, whereas bradykinin (BK) is cleaved with similar efficiency by both domains 7,8. By contrast, the N-domain is solely responsible for the degradation of *N*-acetyl Ser–Asp–Lys–Pro, a peptide that regulates proliferation of haematopoietic stem cells and fibroblasts 9,10. The prevention and reversal of fibrosis in the heart, lung and kidney by nonselective ACE inhibitors has been attributed to protection of *N*-acetyl Ser–Asp–Lys–Pro from degradation in these tissues 11. The recognition that the two domains have physiological domain-selective substrates has provided the impetus for developing domain-selective inhibitors 4,12,13. Many ACE inhibitors have low concentration of inhibitor that reduces enzyme activity by 50% (IC_50_) values for both domains of somatic ACE and this lack of domain specificity has the potential for eliciting side effects resulting from the inhibition of the hydrolysis of bradykinin (BK) and substance-P peptides. There is, therefore, much interest in elucidating the detailed molecular interactions that define domain selectivity in order to provide the basis for structure-based design of ACE inhibitors that selectively target one of the two active sites of mammalian ACE. Substantial information has been gleaned from crystal structures of single-domain forms of human ACE co-crystallized with inhibitors. However, rapid progress in these studies is hindered somewhat by the difficulty in obtaining sufficient protein suitable for crystal growth which necessitates extensive removal of the glycans from the highly glycosylated domains 3,4,12.

These difficulties have been overcome by using a recombinant *Drosophila melanogaster* ACE (AnCE, a single-domain protein with ACE-like activity) as a suitable model for providing valuable structural information on the interaction between synthetic ACE inhibitors and the enzyme active site. AnCE is a single-domain glycosylated protein that closely shares enzymatic properties with human ACE (in particular the C-domain of human somatic ACE) and is inhibited by classic inhibitors of the human enzymes 14–17. Importantly, recombinant AnCE expressed in *Pichia pastoris* readily forms crystals of proteins in complex with inhibitors without the need for removal of sugars 18. Comparison of the structures of *D. melanogaster* AnCE with human ACE in complex with the ACE inhibitors captopril and lisinopril confirmed the close similarity in the binding of inhibitors in the active site cleft 18,19.

In this study, we elucidate how the natural peptides Ang II (the principal end-product of the renin-angiotensin–aldosterone system), Arg–Pro–Pro (the BK-derived peptide) and bradykinin-potentiating peptide-b (BPPb, a snake venom inhibitor) bind to the active site of AnCE, revealing novel interactions involving several enzyme subsites. This information will be of value for the understanding of the current and other related Pro-rich peptides as potent inhibitors of AnCE.

## Results

### Crystal structure of *D. melanogaster* AnCE-peptide complexes

**Fig. 1:**
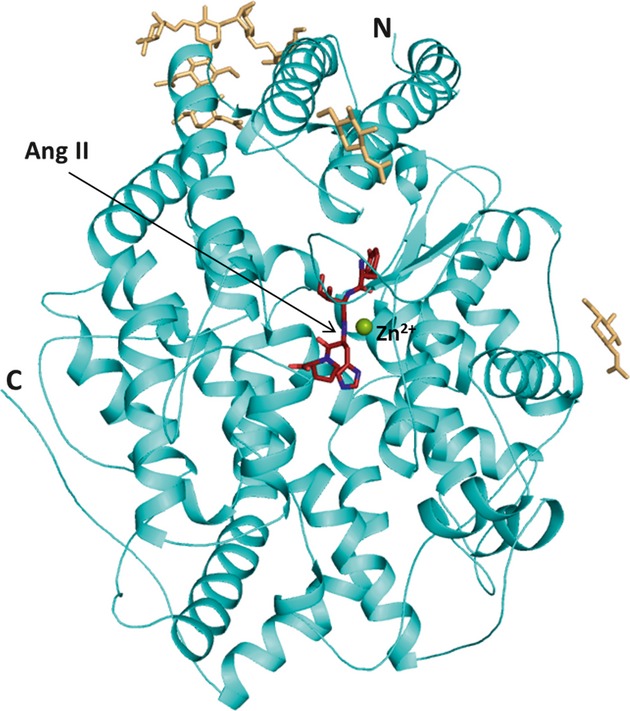
Substrate-bound *Drosophila melanogaster* AnCE crystal structure. AnCE (cyan) in cartoon representation, with Ang II as red sticks, glycosylation carbohydrates as yellow sticks. The catalytic zinc ion is shown as an olive green sphere.

**Fig. 2:**
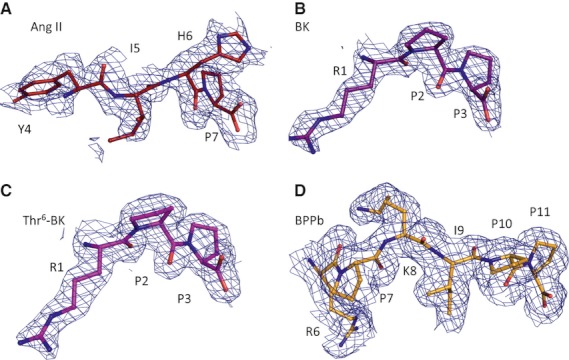
Portions of the difference electron density map for the bound peptide in the active site of AnCE. Electron density map is contoured at 1σ level. The picture was created using a Fourier difference density map in which the peptide atoms were omitted (A) Ang II, (B) BK, (C) Thr^6^–BK and (D) BPPb in the crystal structure of their respective complex with AnCE.

**Fig. 3:**
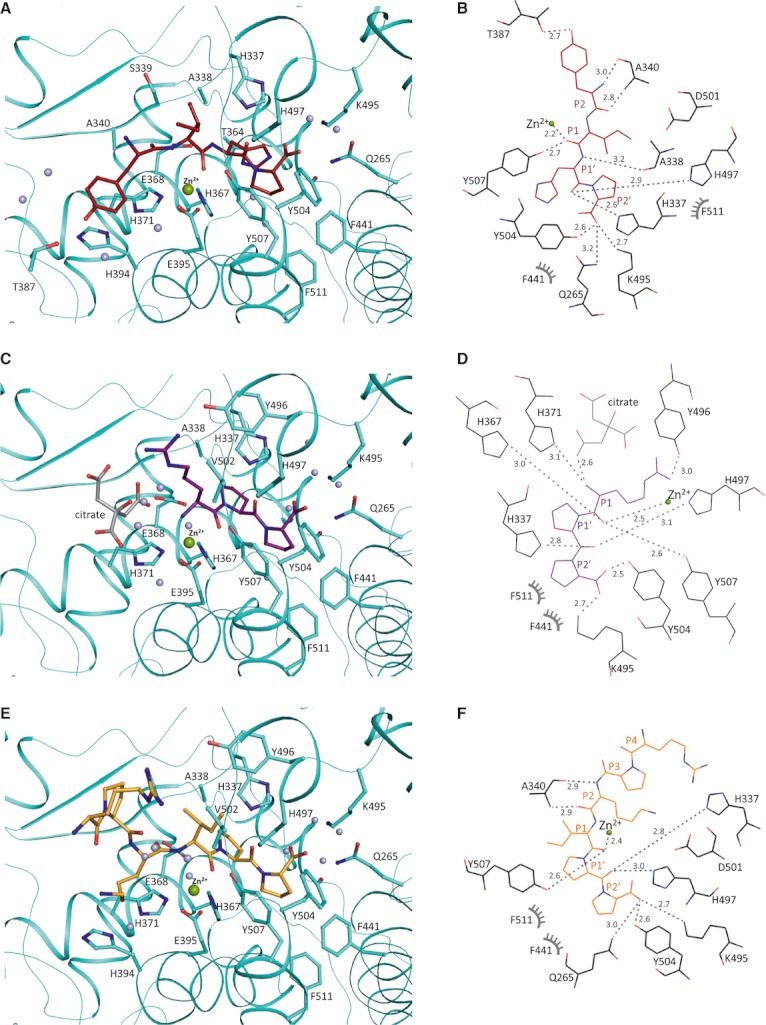
AnCE–peptide interactions. (A) Ang II-bound AnCE crystal structure. AnCE (cyan), with Ang II in red sticks. The catalytic zinc ion is shown as a green sphere. Bound water molecules as small spheres. (B) Schematic view of Ang II binding. Hydrophobic interactions, hydrogen bonds and distances cited (grey). (C) BK-bound AnCE crystal structure. AnCE (cyan), with BK in pink sticks. Citrate ion in grey. (D) Schematic view of BK binding. Hydrophobic interactions, hydrogen bonds and distances cited (grey). (E) BPPb-bound AnCE crystal structure. AnCE (cyan) with BPPb in orange sticks. (F) Schematic view of BPPb binding. Hydrophobic interactions, hydrogen bonds and distances cited (grey).

AnCE was co-crystallized with Ang II, BK, Thr^6^–BK, BPPb and their structures were determined at 2-Å resolution (Fig. 1 and Tables 1 and 2). The co-crystallization of Ang I (Asp–Arg–Val–Tyr–Ile–His–Pro–Phe–His–Leu) with AnCE resulted in conversion to Ang II (Asp–Arg–Val–Tyr–Ile–His–Pro–Phe) which can be observed in the substrate-binding channel. In the AnCE–Ang II peptide complex structure, clear electron density was observed for the tetrapeptide Tyr–Ile–His–Pro (Fig. 2A and Table 2). Ang II is resistant to hydrolysis by AnCE (Fig. S1) and repositions itself in the active site so that the penultimate C-terminal Pro residue shifts from S2 to the S2′ subsite after the hydrolysis of Ang I. Based on molecular modelling, we predict that the C-terminal Phe of Ang II could be accommodated in the binding pocket. It is likely that the side chain of Phe occupies the hydrophobic pocket surrounded by aromatic residues Tyr496, Phe127, Trp263 and Phe169 and the peptide main chain atoms extend into the solvent channel by displacing some of the bound water molecules towards a cluster of polar residues Asp360, Gln266, Asn261 up to Glu269. Unlike Ang II, BK (Arg–Pro–Pro–Gly–Phe–Ser–Pro–Phe–Arg) and Thr^6^–BK (Arg–Pro–Pro–Gly–Phe–Thr–Pro–Phe–Arg) undergo degradation by AnCE to BK1–7 and Thr^6^–BK1–7, respectively and then to BK1–5 (Fig. S2A). BK1–5 is further cleaved by AnCE to release the dipeptide Gly–Phe (Fig. S2B) and therefore under the conditions employed in the crystallization, it is expected that both BK and Thr^6^–BK will be sequentially hydrolysed to the final product, Arg–Pro–Pro (BK1-3). Therefore, it was not surprising that the structures of AnCE in complex with BK and Thr^6^–BK showed Arg–Pro–Pro bound in a similar fashion in the active site cleft (Fig. 2B,C and Tables 2 and 3). For the inhibitory BPPb peptide (pGlu–Gly–Leu–Pro–Pro–Arg–Pro–Lys–Ile–Pro–Pro; *K*_i_, 107 μm) clear continuous electron density was observed for residues Arg–Pro–Lys–Ile–Pro–Pro (Fig. 2D and Table 2). In all four complex structures the catalytic zinc ion at the active site provides the anchor point through direct coordination with the peptide backbone. The peptide interactions with the active site residues are further stabilized by a string of bound water molecules (Fig. 3A,C,E and S3). Optimal interaction of the substrate with residues in the active site leads to the displacement of a water molecule, previously in coordination with the zinc ion, towards the active site Glu. This displacement results in an enhancement of the nucleophilicity of the water molecule and positions it for nucleophilic attack on the substrate carbonyl carbon. The binding of the peptide did not introduce any conformational change in the active site of the protein.

**Table 1:** Crystallographic statistics

	AnCE–Ang II peptide complex	AnCE–BPPb peptide complex	AnCE–BK peptide complex	AnCE–Thr^6^–BK peptide complex
Resolution (Å)	1.99	1.99	1.99	1.99
Space group	R3	R3	R3	R3
Cell dimensions (Å; *a* = *b*, *c*)	173.41, 102.24	173.21, 102.89	173.12, 101.67	173.29, 101.45
Angle (°; α = β, γ)	90, 120	90, 120	90, 120	90, 120
Total/unique reflections	471,847/78,501	413,813/78,895	510,263/89,919	476,736/89,784
Completeness (%)	98.4 (91.9)	90.3 (85.3)	96.6 (82.3)	96.7 (83.2)
*R*_symm_^a^	5.3 (23.2)	4.9 (23.0)	8.8 (42.1)	5.0 (16.9)
*I*/σ (*I*)	19.0 (4)	18.2 (3.5)	9.1 (2.2)	14.9 (5.1)
*R*_cryst_^b^	18.8	20.3	18.1	18.4
*R*_free_^c^	21.0	22.5	19.9	20.0
Rmsd in bond lengths (Å)	0.007	0.007	0.006	0.006
Rmsd in bond angles (°)	0.99	1.03	0.91	0.90
B-factor statistics (Å^2^)				
Protein all atoms	34.3	36.7	28.3	25.4
Protein main chain atoms	34.0	36.4	28.0	25.0
Protein side chain atoms	34.6	37.0	28.5	25.7
Solvent atoms	41.9	41.8	36.1	35.3
Peptide atoms	51.9	40.5	30.9	25.9
Zn^2+^ ion	35.0	55.7	24.6	21.0
Glycosylated carbohydrate atoms	52.7	56.3	40.7	37.3
PDB code	4AA1	4AA2	4ASQ	4ASR

Values in parentheses are for the last resolution shell.

a *R*_symm_ = Σ*h*Σ*i*[|*Ii*(*h*) − <*I*(*h*)>|/Σ*h*Σ*i Ii*(*h*)], where *Ii* is the *i*th measurement and <*I*(*h*)> is the weighted mean of all the measurements of *I*(*h*).

b *R*_cryst_ = Σ*h*|*F*_o_ − *F*_c_|/Σ*hF*_o_, where *F*_o_ and *F*_c_ are observed and calculated structure factor amplitudes of reflection *h*, respectively.

c *R*_free_ is equal to *R*_cryst_ for a randomly selected 5% subset of reflections.

**Table 2:** Substrates bound to *Drosophila melanogaster* AnCE in the crystal structures. Amino acids left after degradation are underlined

Substrate	Peptide sequence	Resolution of the crystal structure (Å)	Ordered visible peptide observed in the structure
Ang II	DRVYIHPF	2.0	YIHP
BK	RPPGFSPFR	2.0	RPP
Thr^6^–BK	RPPGFTPFR	2.0	RPP
BPPb	pyroEGLPPRPKIPP	2.0	RPKIPP

**Table 3:** Hydrogen bond interactions of *Drosophila melanogaster* AnCE with Ang II, BK, Thr^6^–BK and BPPb peptides

Ang II peptide			BPPb peptide			BK peptide			Thr^6^–BK peptide		
			
Ligand atom	Interacting atom from AnCE (and Zn ion)	Distance (Å)	Ligand atom	Interacting atom from AnCE (and Zn ion)	Distance (Å)	Ligand atom	Interacting atom from AnCE (and Zn ion)	Distance (Å)	Ligand atom	Interacting atom from AnCE (and Zn ion)	Distance (Å)
Y4 N	A340 O	3.1	K3 N	A340 O	2.9						
Y4 O	A340 N	2.8	K3 O	A340 N	2.9						
Y4 OH	T387 OG1	2.7									
						R1 O	H367 NE2	3.0	R1 O	H367 NE2	3.1
I5 O	Y507 OH	2.7	I4 O	Y507 OH	2.6	R1 O	Y507 OH	2.6	R1 O	Y507 OH	2.6
I5 O	Zinc ion	2.2	I4 O	Zinc ion	2.4	R1 O	Zinc ion	2.5	R1 O	Zinc ion	2.5
						R1 N	H371 NE2	3.1	R1 N	H371 NE2	3.2
						R1 NH1	Y496 OH	3.0	R1 NH1	Y496 OH	3.0
H6 N	A338 O	3.2									
H6 O	H337 NE2	2.6	P5 O	H337 NE2	2.8	P2 O	H337 NE2	2.7	P2 O	H337 NE2	2.8
H6 O	H497 NE2	2.9	P5 O	H497 NE2	3.0	P2 O	H497 NE2	3.1	P2 O	H497 NE2	3.2
P7 O	Y504 OH	2.6	P6 O	Y504 OH	2.6	P3 O	Y504 OH	2.5	P3 O	Y504 OH	2.5
P7 O	K495 NZ	2.7	P6 O	K495 NZ	2.7	P3 O	K495 NZ	2.7	P3 O	K495 NZ	2.7
P7 O	Q265 NE2	3.2	P6 O	Q265 NE2	3.0						

**Table 4:** Kinetic constants for *Drosophila melanogaster* AnCE. App *K*_m_, apparent or observed *K*_m_; SE, standard error

Substrate	Inhibitor	App *K*_m_ (μm)	*K*_i_ (μm)	SE
Ang I		1040^a^		210
HHL	Ang II^b^		75	9.6
Abz–YRK(Dnp)P	BPPb		107	14

a [17].

b Ang II was not cleaved by AnCE.

In the case of AnCE–Ang II peptide complex, the observable part of the Ang II peptide docked with residues P2, P1, P1′ and P2′, occupying the S2, S1, S1′ and S2′ subsites, respectively (Table 3 and Fig. 3A,B). In this configuration, the peptide could not be cleaved, suggesting that Ang II is a true competitive inhibitor of AnCE-mediated conversion of Ang I. The C-terminal Pro residue (P2′) is clearly visible and anchors the peptide at the S2′ subsite by hydrogen bonds with three residues (Gln265, Tyr504 and Lys495), whereas the pyrrolidine ring is stabilized through hydrophobic interaction with the surrounding aromatic residues (in particular Tyr507 and Phe441). The main chain of the His (P1′) residue from the Ang II peptide makes hydrogen bond interactions with His337 and His497. At the P1 position, the Ile residue is involved in a tetrahedral coordination with the zinc ion and stabilized by a hydrogen bond with Tyr507. The visible electron density terminates at the P2 position with the main chain of Tyr strongly interacting with the main chain of Ala340 (through two hydrogen bonds). The hydroxyl group of Tyr interacts with Thr387 of AnCE.

The detailed structures of AnCE co-crystallized with BK and Thr^6^–BK are presented in Fig. 3C,D and S3A,B and exhibit similar mode of binding for the final cleavage product, BK(1-3). Strong hydrogen bond interactions with AnCE anchor the main chain of the N-terminal Arg (P1) at the S1 site (His367, His371 and Tyr507), which is also involved in coordination with the zinc ion. Furthermore, the side chain for this residue is stabilized through contact with Tyr496. Additionally, both structures bind a citrate ion (from the crystallization medium) at the same position, making contact with the N-terminus of the bound BK (1-3) peptide. The citrate ion also interacts directly with the AnCE main chain and the surrounding water molecules. The two Pro residues at positions P1′ and P2′ form a strong interaction with AnCE through hydrogen bonds with residues His497, His337 and Tyr504, Lys495 respectively. The general mode of binding for BK (1-3) appears similar to that of Ang II, particularly through the various hydrogen bonds with AnCE. Experimental evidence suggests a preferred affinity for Pro at the S2′ binding site and highlights the importance of the hydrophobic pocket (formed by residues Phe441, Phe511, Tyr504 and Tyr507) for peptide recognition, which allows the products of BK and Thr^6^–BK hydrolysis to shift position and ‘register’ in the active site subsites.

The structure of the AnCE–BPPb complex revealed interactions between the pyrrolidine ring of the two Pro residues in the penultimate and C-terminal positions (P1′ and P2′, respectively) and the amino acid side chains that form the S1′ and S2′ enzyme subsites, respectively (Table 3 and Fig. 3E,F). A cluster of aromatic residues forms this binding pocket (Phe441, Phe511, Tyr504, Tyr507), and Tyr507 ‘stacks’ against the C-terminal Pro, thus enhancing the interaction with AnCE. The two Pro residues (P1′ and P2′) are strongly anchored in the prime binding sites (S1′ and S2′) by hydrogen bonding with multiple residues. The P2′ Pro, in particular, has strong interactions with Gln265, Tyr504 and Lys495. The Ile residue from the BPPb peptide forms a direct coordination with the catalytic zinc ion by replacing the usual water molecule observed in the native AnCE structure 18. This residue is further stabilized by interaction with the hydroxyl group of Tyr507. The main chain of the next Lys residue of the peptide in the P2 position interacts with Ala340 through two hydrogen bonds. Of the two visible N-terminal residues of BPPb, namely Pro and Arg, Pro is stabilized by hydrophobic interaction with a bulky Trp341 residue, whereas the Arg side chain forms a weak interaction with AnCE via a water-mediated hydrogen bond with Asp501. This part of the structure shows a clear solvent network involving many charged residues and provides room for the accommodation of longer peptide substrates. The homology between AnCE and ACE, and the presence of ‘unique’ N- or C-domain residues provides opportunities for these subsites to be exploited further for the rational design of new domain-selective ACE inhibitors.

Comparison of the four structures clearly shows a common mode of peptide binding to AnCE. First, the P2′ Pro appears as an essential element in the binding with strong hydrogen bonds and hydrophobic interactions at the S2′ subsite. Second, the backbone interactions at the P1′ and P1 sites are identical in all structures with His337 and His497 stabilizing P1′, and Tyr507 and the zinc ion interacting with the P1 main chain. Finally, Ala340 stabilizes the main chain of P2 when the peptides are visible at that position. This arrangement in the orientation of the peptide backbone makes the P1–P1′ linkage resistant to cleavage.

The common mode of recognition in the four complex structures involves the main chain of the peptide taking control at the catalytic site by replacing a water molecule involved in the proteolysis mechanism. Furthermore, Glu368 (potentially acting as the catalytic base that protonates the amine product) does not seem to be involved in any peptide interaction. Altogether, the peptides presented here show strong interactions with AnCE via their backbone atoms and through direct coordination with the zinc ion, thus preventing proteolysis.

### Ang II is a competitive inhibitor of *D. melanogaster* AnCE

**Fig. 4:**
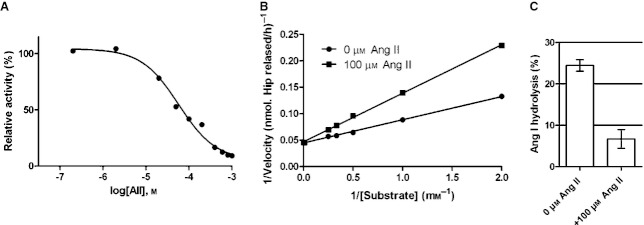
Inhibition of *Drosophila melanogaster* AnCE by Ang II. AnCE was assayed using HHL as substrate in presence of various concentrations of Ang II as described in the Experimental procedures. (A) Data are expressed as a percentage of uninhibited activity and each data point is the mean of three replicates. (B) Lineweaver–Burk plot showing the competitive nature of the inhibition by Ang II of the hydrolysis of HHL by AnCE. (C) The effect of Ang II on the hydrolysis of Ang I (100 μm) by AnCE was determined by quantifying the amount of Ang I consumed in the reaction in the absence and presence of 100 μm Ang II. The results are expressed as % hydrolysis occurring in 30 min and are means ± SEM (*n* = 4).

The crystal structure of AnCE in complex with Ang II provided direct structural evidence for Ang II being a competitive inhibitor of AnCE activity. We sought to confirm this with biochemical data using the synthetic substrate hippuryl–histidyl–leucine (HHL) to assay the peptidyl dipeptidase activity of AnCE. These experiments clearly showed that the hydrolysis of HHL by AnCE was inhibited by Ang II with an IC_50_ of 58 μm (Fig. 4A) and that Ang II was a competitive inhibitor with a *K*_i_ value of 76 μm (Fig. 4B). Consistent with these data, Ang II strongly inhibited the conversion of Ang I to Ang II (73% inhibition) when equimolar concentrations of Ang I and Ang II were incubated with recombinant AnCE (Fig. 4C).

## Discussion

**Fig. 5:**
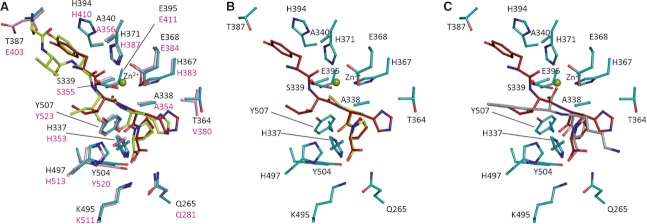
Comparison of AnCE–Ang II interactions. (A) Comparison of Ang II binding in AnCE and the C-domain human somatic ACE crystal structures. AnCE (cyan)–Ang II (red) complex superposed with human ACE (pink)–Ang II (green) complex (PDB: 4APH) 1. The catalytic zinc ion is shown as a green sphere. Residue labels are in black and pink for AnCE and human ACE proteins respectively. (B) Comparison of Ang II and captopril binding in AnCE. AnCE (cyan)–Ang II (red) complex superposed with AnCE–captopril (yellow) complex (PDB: 2X8Z) [18]. (C) Comparison of Ang II and lisinopril binding in AnCE. AnCE (cyan)–Ang II (red) complex superposed with AnCE–lisinopril (grey) complex (PDB: 2X91) [18].

We report the molecular structures of natural peptides in complex with an AnCE protein. BPPb is one of several blood-pressure-lowering peptides isolated from the venom of *Bothrops jararaca* and has been shown to be an inhibitor of *D. melanogaster* AnCE and both domains of human somatic ACE, but with > 200-fold selectivity towards the C-domain. BPPb is a relatively weak inhibitor of AnCE, displaying a *K*_i_ that is one and three orders of magnitude greater than the dissociation constants recorded for the human N-domain and C-domain ACE, respectively. Nevertheless, the structure of AnCE in complex with BPPb has provided detailed molecular information on how the venom peptide inhibits ACE and causes hypotension and shock in envenomated prey. BPPb has a proline as the penultimate C-terminal residue, a feature shared with several other BK-potentiating peptides found in the venom. The cyclic nature of the proline side chain restricts free rotation around the Cα–N bond and the lack of a N–hydrogen bond prevents the formation of H bonds with carbonyls. These structural properties, unique amongst the natural amino acids, protect Ang II and BPPb from peptidase cleavage of the peptide bond that incorporates the amino group of proline 20. These observations are consistent with the experimental results gleaned from the structural studies of AnCE co-crystallized with BK and Thr^6^–BK. In both cases, complete hydrolysis of the peptide to the end point resulted in clear visible electron density for the tripeptide (Arg–Pro–Pro) in their respective crystal structures. Importantly, the general mode of natural peptide inhibitor binding to AnCE is shared with human ACE. In a recent detailed report 21, we provided the molecular basis for the regulation of human ACE activity by natural inhibitory peptides Ang II and BPPb. In that report, we also highlighted the role of the proline residue, peptide backbone recognition and coordination with the zinc ion in the binding of Ang II to the C-terminal domain of human somatic ACE (Fig. 5A). This might explain the success of clinically used small molecule ACE inhibitors, such as captopril and lisinopril, that are proline derivatives and are able to interact directly with the zinc ion (Fig. 5B,C), thus mimicking the natural peptide inhibitors.

*Drosophila melanogaster* AnCE, like other members of the ACE family in insects, is a highly promiscuous peptidase and converts Ang I to Ang II and inactivates BK, even though these peptides are not found in flies 17. The broad specificity of the insect enzyme might therefore reflect a general metabolic role for the carboxydipeptidase in peptide metabolism in animals ancestral to the vertebrates before the need for an angiotensin-processing enzyme as part of the renin-angiotensin–aldosterone system. In turn, products of promiscuous proteases often become competing substrates until no more susceptible peptide bonds remain. In the case of Ang II, the proline residue in the penultimate C-terminal position confers resistance to further cleavage by AnCE and mammalian ACE, ensuring that Ang II is the principal product of angiotensinogen metabolism. The Pro at position 7 is also important for binding to the Ang II type 1 (AT_1_) receptor, the principal mediator of Ang II actions in mammals, and is absolutely conserved in all vertebrate Ang II peptides characterized to date 22.

In summary, we have elucidated the molecular interactions that underpin the inhibitory properties of four natural peptides towards AnCE. The work reported here provides new structural information of value in understanding the substrate and inhibitor specificity of the ACE family of peptidases and increasing our understanding of its fascinating evolution from a single- to a two-catalytic-domain enzyme.

## Experimental procedures

All chemicals including BPPb, BK, Thr^6^–BK, Phe-Gly and Ang I peptides were purchased from Sigma-Aldrich (Gillingham, UK). *Drosophila melanogaster* AnCE was generated by expression in *P. pastoris* and purified by hydrophobic interaction chromatography and size-exclusion chromatography to homogeneity, as described previously 18.

### Assay of AnCE activity

This was determined in a reaction buffer comprising 0.1 m Hepes buffer, pH 7.5, 10 μm ZnSO_4_, 0.15 m NaCl and either HHL (5 mm, final concentration) or Ang I (100 μm, final concentration) as the substrate. HPLC with UV-detection (214 nm) was employed to quantify the release of hippuric acid from the hydrolysis of HHL and the conversion of Ang I to Ang II, as described previously 16,23. When Ang II was tested as an inhibitor of Ang I conversion to Ang II, AnCE activity was measured by comparing the decline in Ang I in the absence and presence of an initial concentration of 100 μm Ang II. Recombinant AnCE was diluted in reaction buffer containing 0.1 mg·mL^−1^ of BSA to give final concentrations of 250 ng·μL^−1^. To determine the *K*_i_ of BPPb, AnCE activity was assayed using Abz-YRK(Dnp)P as substrate, as described by Bingham *et al*. 2006.

### X-Ray crystallography

Native AnCE was preincubated with BK, Thr^6^–BK, BPPb or Ang I peptide (protein/peptide molar ratio of 1 : 14) on ice for 5 h before crystallization. Co-crystals were obtained with 2 μL of the AnCE–peptide sample (10 mg·mL^−1^ in 5 mm Hepes pH 7.5, 0.1 mm phenylmethanesulfonyl fluoride and 10 μm zinc acetate) mixed with an equal volume of reservoir solution (100 mm Hepes pH 7.5 and 1.3 m sodium citrate) and suspended above the well as a hanging drop. Diffraction-quality crystals of AnCE–peptide complex appeared after ∼ 1 week.

X-Ray diffraction data for AnCE–peptide complexes were collected on PX stations IO2 and IO3 at Diamond Light Source (Didcot, UK). No cryoprotectant was used to keep the crystal at constant temperature under the liquid nitrogen jet during data collection. For each complex, 100 images were collected by using a Quantum-4 CCD detector (ADSC Systems, Poway, CA, USA). Raw data images were indexed and scaled using the hkl2000 software package 24. Data reduction was carried out by using the CCP4 program truncate 25. Initial phases for structure solution were obtained using the molecular replacement routines of the phaser program 26. The atomic coordinates of native AnCE (PDB: 2X8Y 18) were used as a search model for AnCE–peptide complexes. The resultant models were refined using refmac5 27. Five per cent of reflections were separated as the *R*_free_ set and used for cross-validation 28. Manual adjustments of the model were carried out using coot 29. Water molecules were added at positions where *F*_o_ − *F*_c_ electron density peaks exceeded 3σ and potential hydrogen bonds could be made. Based on visible electron-density interpretation, BK/Thr^6^–BK/BPPb/Ang II peptide was incorporated in the structure and further refinement was carried out. Validation was conducted with the aid of molprobity 30. There were no residues in the disallowed region of the Ramachandran plot. Crystallographic data statistics are summarized in Table 1. All figures were drawn using pymol (DeLano Scientific LLC, San Carlos, CA, USA) and rendered with pov-ray. Hydrogen bonds were verified using hbplus 31.

## Abbreviations

ACE: angiotensin-1 converting enzyme
AnCE: an ACE homologue from *Drosophila melanogaster*
Ang I: angiotensin I
Ang II: angiotensin II
BK: bradykinin
BPPb: bradykinin-potentiating peptide-b from snake venom
HHL: hippuryl–histidyl–leucine

## Supporting information

**Fig. S1.** The conversion of Ang I to Ang II (A, B) and the stability of Ang II (C, D) in the presence of recombinant AnCE.

**Fig. S2.** Sequential cleavage of Thr^6^–BK by AnCE.

**Fig. S3.** (A) Thr^6^–BK-bound AnCE crystal structure. AnCE (cyan), with Thr^6^–BK in magenta sticks. Citrate ion in grey. (B) Schematic view of Thr^6^–BK. Binding with hydrophobic interactions, hydrogen bonds and distances cited (grey).
