# Supplementary material for: Structural basis of peptide recognition by the angiotensin-1 converting enzyme homologue AnCE from Drosophila melanogaster
Source: FEBS J. 2012 Nov 22;279(24):4525–34. doi: 10.1111/febs.12038 (PMC3564407; doi:10.1111/febs.12038)

the FEBS Journal

www.febsjournal.org

Structural basis of peptide recognition by the angiotensin-1 converting enzyme homologue AnCE from *Drosophila melanogaster*

Mohd Akif, Geoffrey Masuyer, Richard J. Bingham, Edward D. Sturrock, R. Elwyn Isaac and K. Ravi Acharya

DOI: 10.1111/febs.12038

Supplementary information

Structural Basis of Substrate Recognition by the Angiotensin-I Converting Enzyme Homologue AnCE from *Drosophila melanogaster*

Mohd Akif, Geoffrey Masuyer, Richard J. Bingham, Edward D. Sturrock, R. Elwyn Isaac and K. Ravi Acharya

Figure S1

The conversion of Ang I to Ang II (A and B) and the stability of Ang II (C and D) in the presence of recombinant AnCE.

Peptides (50 µM) were incubated with 3 µg of AnCE in 0.1 M HEPES buffer, pH 7.5, 10 µM $ZnSO_4$ and 0.15 M NaCl. After 4h at 25°C, the enzyme activity was terminated by adding 8 % trifluoroacetic acid (20 µl) before HPLC analysis [17]. The peptide elution was monitored using a u.v. detector set at 214 nm.

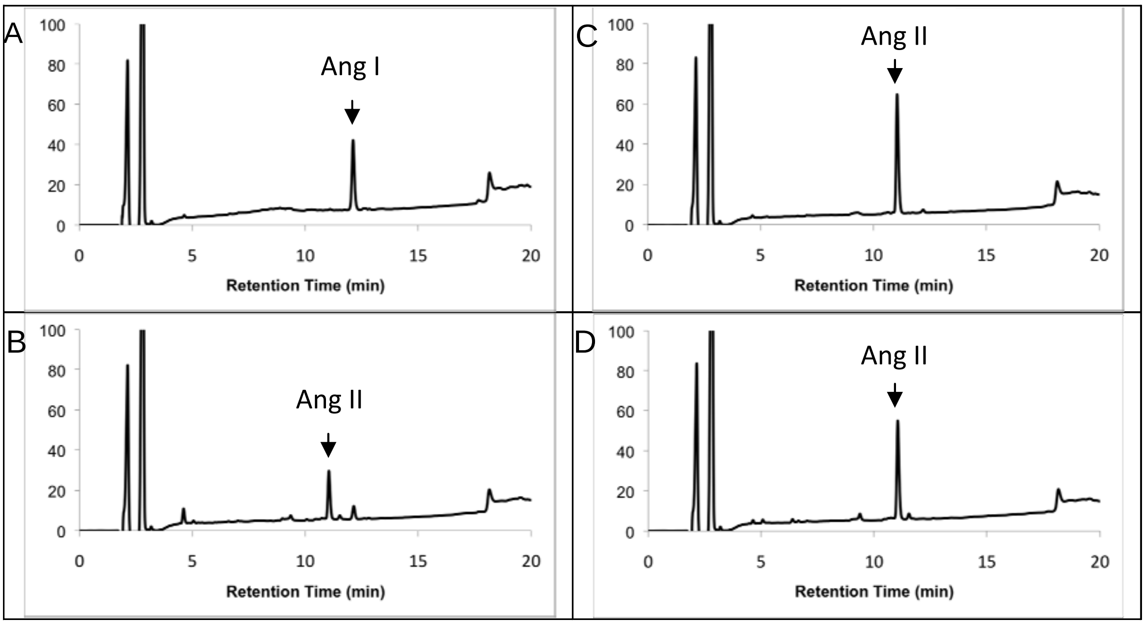

Figure S2

Sequential cleavage of Thr^6-BK by AnCE.

(A). Thr^6-BK was incubated with AnCE (50 ng) and the peptide products were separated by HPLC as described previously [17]. Hydrolysis products were identified by comparison of their retention times with synthetic peptides. 1, Thr-Pro;2, Phe-Arg; 3, BK 1-5; 4, Thr^6-BK 1-7. **(B).** BK 1-5 was incubated with 3 µg of AnCE in 0.1 M HEPES buffer, pH 7.5, 10 µM $ZnSO_4$ and 0.15 M NaCl. After 4h at 25°C, the enzyme activity was terminated by adding 8 % trifluoroacetic acid (20 µl) before HPLC analysis [17]. The C-terminal dipeptide Gly-Phe was identified by its retention time relative to synthetic material.

A

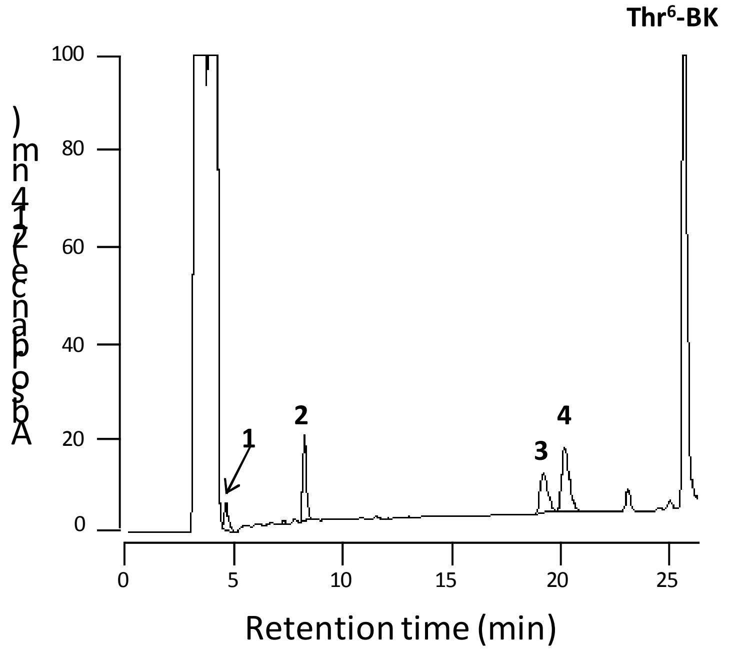

B

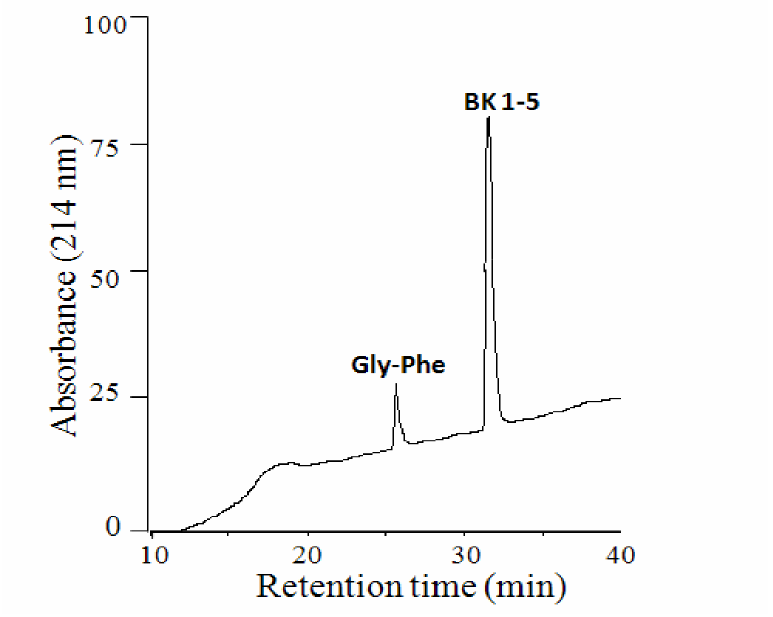

Figure S3

(**A**) **Thr6-BK - bound AnCE crystal structure.** AnCE (cyan), with Thr6-BK in magenta sticks. Citrate ion in grey. (**B**) **Schematic view of Thr6-BK.** Binding with hydrophobic interactions, hydrogen bonds and distances cited (grey).

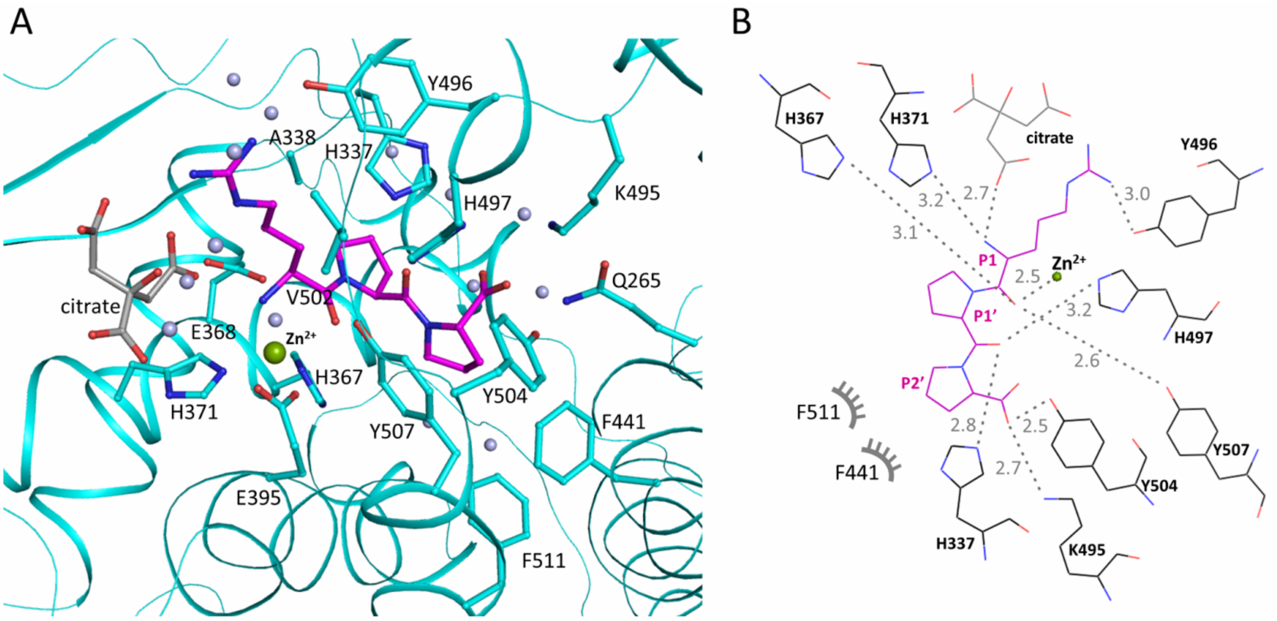

Supplement: Supplementary file 1 [file febs0279-4525-SD1.zip › febs12038-sup-0001-FigS1-S3.pdf]
